# Characteristics and comparison between diabetes mellitus and non-diabetes mellitus among chronic kidney disease patients: A cross-sectional study of the Chinese Cohort Study of Chronic Kidney Disease (C-STRIDE)

**DOI:** 10.18632/oncotarget.22368

**Published:** 2017-11-10

**Authors:** Jun-Jun Zhang, Liu Yang, Jun-Wen Huang, Yu-Jie Liu, Jin-Wei Wang, Lu-Xia Zhang, Ming-Hui Zhao, Zhang-Suo Liu

**Affiliations:** ^1^ Department of Nephrology, The First Affiliated Hospital of Zhengzhou University, Zhengzhou, Henan, China; ^2^ Research Institute of Nephropathy, Zhengzhou University, Zhengzhou, Henan, China; ^3^ Department of Nephrology and Rheumatology, Children’s Hospital of Zhengzhou City, Zhengzhou, Henan, China; ^4^ Renal Division, Department of Medicine, Peking University First Hospital, Beijing, China

**Keywords:** chronic kidney disease, diabetes mellitus, clinical traits, medication, diabetic nephropathy

## Abstract

Although the prevalence of chronic kidney disease (CKD) and diabetes mellitus (DM) is increasing globally, information on Chinese CKD patients with DM is lacking. A total of 3499 pre-dialysis CKD patients from across China were enrolled in the Chinese Cohort Study of Chronic Kidney Disease (C-STRIDE) between November 2011 and April 2016. We divided the C-STRIDE patients into CKD with DM and CKD without DM groups and compared their clinical, demographic, and laboratory data in this cross-sectional study. CKD patients with DM were older, had a higher male-to-female ratio, and had more complications than CKD patients without DM. Age, smoking, and 24-h urinary protein levels were associated with co-occurrence of CKD and DM. Less than 50% of patients in either group took antilipemic, cardiovascular, cerebrovascular, or anti-anemic drugs. In addition, only 18.38% of CKD patients with DM had undergone a renal biopsy, and diabetic nephropathy was confirmed in 35.4% of them. Our findings suggest that several types of medication and renal biopsies should be used more frequently in the treatment of Chinese CKD patients with DM.

## INTRODUCTION

Incidences of diabetes mellitus (DM) have increased rapidly around the world because of changes in human environments, behaviors, and lifestyles [1–3]. The *International Diabetes Federation Diabetes Atlas* (7th edition) reports that 415 million people suffered from DM in 2015. By 2040, an estimated 642 million people will have DM globally, which equates to approximately 1 in 10 adults. In mainland China, 109.6 million adults have DM; China had more adults with DM than any other countries or territories in the world in 2015 [1]. In addition, an estimated 119.5 million people in China have chronic kidney disease (CKD) [4, 5], and at least 24.3 million Chinese CKD patients also have DM [6]. When a patient has both CKD and DM, the two diseases aggravate each other and result in particularly difficult-to-treat clinical manifestations. However, the clinical and pathological characteristics of Chinese patients with both CKD and DM remain unclear. Here, we collected and analyzed data from an established national pre-dialysis CKD patient cohort, the Chinese Cohort Study of Chronic Kidney Disease (C-STRIDE) [7]. We examined demographic details, clinical characteristics, complications, concomitant medication, and histopathological diagnosis of patients with both CKD and DM compared to patients with CKD alone in an effort to identify the most effective clinical treatment strategies.

## RESULTS

### Demographic, clinical, and laboratory characteristics of CKD patients with and without DM

A total of 3499 pre-dialysis CKD patients were included in the study, of which 2066 were males (59.05%) and 1433 were females (40.95%) (Table 1). Of these patients, 635 also had DM (CKD with DM, 18.14%) while 2864 did not have DM (CKD without DM, 81.86%).

**Table 1:** Demographic, clinical, and laboratory features of CKD patients with and without DM

	CKD with DM group (*n* = 635)	CKD without DM group (*n* = 2864)	*P* value	Missing number
**Age**				0
Mean age	56.93 ± 10.42	46.20 ± 13.46	<0.001^*^	
18–44 years	83 (13.07%)	1347 (47.10%)	<0.001^*^	
45–59 years	261 (41.10%)	961 (33.60%)		
≥60 years	291 (45.83%)	552 (19.30%)		
**Gender**			<0.001^*^	0
Male	412 (64.88%)	1654 (57.75%)		
Female	223 (35.12%)	1210 (42.25%)		
**Education completed**			<0.001^*^	35
Junior high school or less	348 (55.59%)	1197 (42.18%)		
Senior high school and above	278 (44.41%)	1641 (57.82%)		
**BMI**	25.44 ± 3.39	24.29 ± 3.64	<0.001^*^	329
**Systolic pressure (mmHg)**	137.86 ± 18.19	127.65 ± 16.81	<0.001^*^	443
**Diastolic pressure (mmHg)**	80.72 ± 10.53	81.08 ± 11.83	0.85	443
**CKD stage**			<0.001^*^	294
CKD 1	31 (5.35%)	418 (15.92%)		
CKD 2	48 (8.29%)	483 (18.39%)		
CKD 3a	110 (19.00%)	392 (14.93%)		
CKD 3b	180 (31.09%)	601 (22.89%)		
CKD 4	210 (36.27%)	732 (27.88%)		
**Smoking history**			<0.001^*^	110
Yes	300 (48.86%)	1005 (36.22%)		
**Frequency of alcohol use in past year**			0.13	136
Never	499 (81.67%)	2151 (78.16%)		
Occasionally	84 (13.75%)	467 (16.97%)		
Frequently	28 (4.58%)	134 (4.87%)		
**Previous medical illness**				
Hypertension	515 (81.10%)	1728 (61.10%)	<0.001^*^	36
Myocardial infarction	23 (3.62%)	49 (1.73%)	0.003^*^	36
Congestive heart failure	10 (1.58%)	22 (0.78%)	0.06	41
Arrhythmia	42 (6.64%)	96 (3.39%)	<0.001^*^	38
Cerebrovascular disease	91 (14.40%)	150 (5.31%)	<0.001^*^	40
Peripheral arterial disease	29 (4.59%)	24 (0.85%)	<0.001^*^	39
Malignant tumor	7 (1.11%)	27 (0.95%)	0.72	39
Urologicsurgical procedures	26 (4.15%)	98 (3.50%)	0.43	72
**Laboratory features**				
24-hour urinary protein (g/24h)	1.87 (0.60,4.55)	0.84 (0.31,1.97)	<0.001^*^	589
C-reactive protein (mg/L)	1.43 (0.54,3.46)	1.31 (0.52,3.18)	0.22	662
Hemoglobin (g/L)	118.63 ± 22.70	146.20 ± 13.46	<0.001^*^	381
Total protein (g/L)	51.25 ± 26.71	56.76 ± 25.27	<0.001^*^	438
Serum albumin (g/L)	36.19 ± 7.85	39.22 ± 6.62	<0.001^*^	481
Alkaline phosphatase (µ/L)	72 (56,88)	64 (52,80)	<0.001^*^	492
Serum creatinine (µM)	157.9 (126,218)	142 (98,204)	<0.001^*^	294
Blood uric acid (µM)	403.48 ± 106.49	406.42 ± 121.86	0.42	336
Total cholesterol (mM)	4.68 (3.8,5.9)	4.76 (3.94,5.8)	0.02^*^	478
Triglyceride (mM)	1.86 (1.35,2.67)	1.77 (1.24,2.59)	<0.001^*^	480
Fasting glucose (mM)	6.17 (5.05,7.61)	4.83 (4.38,5.32)	<0.001^*^	507
Low density lipoprotein (mM)	2.56 (2.06,3.32)	2.57 (2.05,3.21)	0.46	528
High density lipoprotein (mM)	1.03 (0.85,1.26)	1.08 (0.9,1.32)	<0.001^*^	528

^*^P < 0.05. BMI: Body Mass Index, CKD: Chronic Kidney Disease.

Male-to-female ratio and mean age were higher in the CKD with DM group. In addition, a larger proportion of CKD patients with DM were smokers (48.86%) compared to CKD patients without DM (36.22%, *P* < 0.001). Percentages of patients who used alcohol did not differ between the groups. CKD patients with DM were generally less well-educated.

CKD patients with DM had more severe kidney disease as indicated by CKD stages (Table 1). Systolic blood pressure was also higher in CKD patients with DM than in those without DM (*P* < 0.001). Diastolic blood pressure was similar in both groups. Mean BMI was higher in CKD patients with DM (25.44 ± 3.39) than in those without DM (24.29 ± 3.64, *P* < 0.001). CKD patients with DM were also more likely to have a history of hypertension, myocardial infarction, arrhythmia, cerebrovascular disease, and peripheral artery disease (*P* < 0.005).

The main laboratory findings are shown in Table 1.Notably, laboratory data suggested that CKD patients with DM had more complicated and severe disease in many ways. Fasting glucose, 24-h urinary protein, serum creatinine, alkaline phosphatase, and triglyceride levels were higher in CKD patients with DM than in those without DM (*P* < 0.001). Hemoglobin, total protein, serum albumin, and high-density lipoprotein cholesterol levels were lower in CKD patients with DM than in those without DM (*P* < 0.001). Total cholesterol was lower in CKD patients with DM than in those without DM (*P* < 0.05).

**Figure 1:**
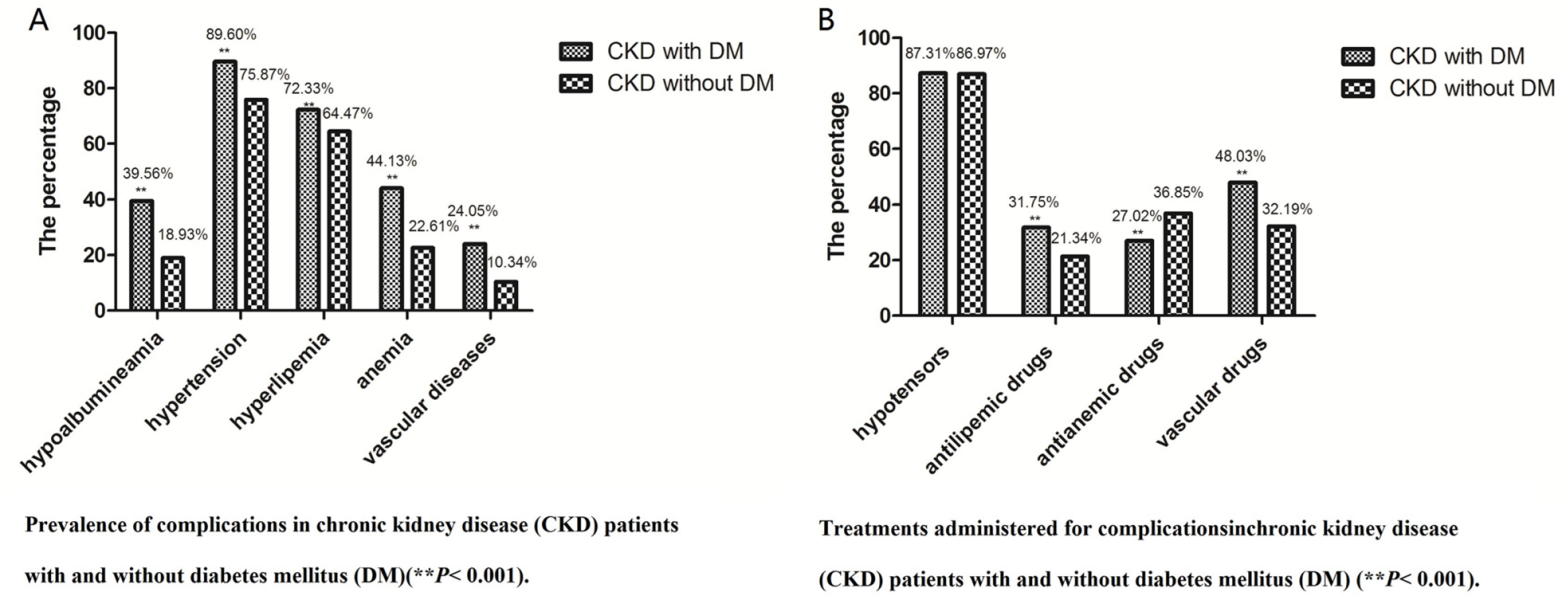
(**A**) Prevalence of complications in chronic kidney disease (CKD) patients with and without diabetes mellitus (DM) (**P < 0.001). (**B)** Treatments administered for complications in chronic kidney disease (CKD) patients with and without diabetes mellitus (DM) (**P < 0.001).

Based on standard definitions, we found that higher percentages of CKD patients with DM had hypertension, hyperlipidemia, anemia, hypoalbuminemia, and vascular disease compared to those without DM (*P* < 0.001) (Figure 1A).

When administration of therapeutic drugs was examined, less than 50% of patients in both groups received antilipemic drugs, cardiovascular drugs, cerebrovascular drugs, and antianemic drugs; only antihypertensive drugs were administered to more than 50% of patients. Although anemia was more common in CKD patients with DM, they received antianemic drugs less often than CKD patients without DM (*P* < 0.001)(Figure 1B).

### Logistic regression analysis of factors related to CKD with and without DM

Multivariable logistic regression analysis revealed that age, 24-h urinary protein, alkaline phosphatase, CKD-3a and CKD-3b stages, BMI, hypoalbuminemia, anemia, hypertension, smoking, and vascular disease history were positively associated with CKD with DM. However, level of education, C-reactive protein, and hyperuricemia were negatively associated with CKD with DM. Gender, CKD-2 and CKD-4 stages, hyperlipidemia, malignant tumor, urologic surgical procedures, frequency of alcohol use, and total protein were not associated with CKD with DM (Table 2).

**Table 2:** Variable assignment table

Variable	Assignment				
CKD Patients (Y)	CKD without DM = 0	CKD with DM = 1			
Gender (X1)	Male = 0	Female = 1			
Age (X2)	18–44 = 0	45–59 = 1	≥60 = 2		
Education completed (X3)	Junior high school or less=0	Senior high school and above=1			
24 h UP (X4)	24 h UP < 1 = 0	1 < 24 h UP < 3.5 = 1	3.5 < 24 h UP < 6 = 2	24 h UP ≥ 6 = 3	
CKD stage (X5)	CKD 1 = 0	CKD 2 = 1	CKD 3a = 2	CKD 3b = 3	CKD 4 = 4
BMI (X6)	BMI < 18.5 = 0	18.5 < BMI < 25 = 1	25 < BMI< 30 = 2	BMI ≥ 30 = 3	
Hypoalbuminemia (X7)	No = 0	Yes = 1			
Hyperlipidemia (X8)	No = 0	Yes = 1			
Anemia (X9)	No = 0	Yes = 1			
Hyperuricemia (X10)	No = 0	Yes = 1			
Hypertension (X11)	No = 0	Yes = 1			
History of cardiovascular diseases (X12)	No = 0	Yes = 1			
Malignant tumor (X13)	No = 0	Yes = 1			
Urologic surgical procedures (X14)	No = 0	Yes = 1			
Smoking history (X15)	No = 0	Yes = 1			
Frequency of alcohol use in past year (X16)	Never = 0	Occasionally = 1	Frequently = 2		
C-reactive protein (X17)	Interquartile of CKD without DM = 0	Interquartile of CKD with DM = 1			
Total protein (X18)	Mean value of CKD without DM = 0	Mean value of CKD with DM = 1			
Alkaline phosphatase (X19)	Interquartile of CKD without DM = 0	Interquartile of CKD with DM = 1			

**Table 3:** Logistic regression analysis for factors associated with CKD with DM

	Univariable analysis		Multivariable analysis	
	OR (95% CI)	*P*-value	OR (95% CI)	*P*-value
**Female** (vs. male)	0.74 (0.62–0.89)	NA	0.86 (0.66–1.12)	0.26
**Age group** (years)				
18–44	Reference		Reference	
45–59	4.41 (3.40–5.73)	NA	3.59 (2.71–4.77)	<0.001^*^
≥60	8.57 (6.59–11.15)	NA	6.60 (4.88–8.92)	<0.001^*^
**Senior high school and above** (vs. junior high school and below)	0.59 (0.49–0.70)	<0.001^*^	0.81 (0.66–0.99)	0.04^*^
**24 h UP** (g/24 h)				
24 h UP <1	Reference		Reference	
1 < 24 h UP < 3.5	1.42 (1.16–1.75)	<0.001^*^	1.37 (1.08–1.74)	0.01^*^
3.5 < 24 h UP < 6	2.88 (2.11–3.93)	<0.001^*^	2.30 (1.61–3.29)	<0.001^*^
24 h UP ≥ 6	4.42 (3.21–6.08)	<0.001^*^	3.54 (2.41–5.19)	<0.001^*^
**C–reactive protein** (mg/L)	1.03 (0.97–1.10)	0.33	0.92 (0.86–1.00)	0.04^*^
**Total protein** (g/L)	0.993 (0.989–0.996)	<0.001^*^	0.997 (0.993–1.002)	0.23
**Alkaline phosphatase** (U/L)	1.63 (1.28–2.08)	<0.001^*^	1.44 (1.08–1.91)	0.01^*^
**CKD stage**				
CKD 1	Reference		Reference	
CKD 2	1.30 (0.81–2.09)	0.28	1.02 (0.62–1.67)	0.95
CKD 3a	3.40 (2.18–5.31)	<0.001^*^	2.26 (1.39–3.66)	0.001^*^
CKD 3b	3.78 (2.52–5.67)	<0.001^*^	1.99 (1.25–3.16)	0.004^*^
CKD 4	3.63 (2.41–5.46)	<0.001^*^	1.62 (0.99–2.65)	0.05
**BMI** (kg/m^2^)				
BMI < 18.5	Reference		Reference	
18.5 ≤ BMI < 25	2.34 (1.08–5.10)	0.03^*^	2.37 (1.01–5.58)	0.048^*^
25 ≤ BMI < 30	4.08 (1.89–8.80)	<0.001^*^	3.85 (1.62–9.14)	0.002^*^
BMI ≥ 30	3.70 (1.67–8.21)	0.001^*^	4.19 (1.73–10.15)	0.002^*^
**Hypoalbuminemia** (yes vs. no)	2.43 (2.01–2.93)	<0.001^*^	2.01 (1.56–2.59)	<0.001^*^
**Hyperlipidemia** (yes vs. no)	1.32 (1.09–1.61)	0.005^*^	1.05 (0.84–1.32)	0.67
**Anemia** (yes vs. no)	2.47 (2.05–2.98)	<0.001^*^	1.73 (1.35–2.22)	<0.001^*^
**Hyperuricemia** (yes vs. no)	0.91 (0.75–1.09)	0.3	0.74 (0.59–0.93)	0.01^*^
**Hypertension** (yes vs. no)	1.82 (1.45–2.28)	<0.001^*^	1.56 (1.01–2.42)	0.04^*^
**History of cardiovascular diseases** (yes vs. no)	2.75 (2.21–3.43)	<0.001^*^	1.57 (1.22–2.01)	<0.001^*^
**Malignant tumor** (yes vs. no)	0.86 (0.37–1.99)	0.73	1.22 (0.48–3.09)	0.68
**Urologic surgical procedures** (yes vs. no)	0.86 (0.55–1.33)	0.49	1.04 (0.63–1.70)	0.88
**Smoking history** (yes vs. no)	1.67 (1.40–1.98)	<0.001^*^	1.41 (1.08–1.83)	0.01^*^
**Frequency of alcohol use in past year**				
Never	Reference		Reference	
Occasionally	0.78 (0.60–1.01)	0.05	0.78 (0.57–1.05)	0.1
Frequently	0.92 (0.60–1.41)	0.7	0.71 (0.42–1.18)	0.18

^*^P < 0.05. BMI: Body Mass Index, CKD: Chronic Kidney Disease, 24 h UP: 24–hour Urinary Protein.

### Renal biopsy and pathological characteristics of CKD patients with and without DM

Of the 635 CKD patients with DM, 111 underwent renal biopsies (18.38%), while 1180 (42.91%) of the 2864 CKD patients without DM underwent a renal biopsies; this difference was significant (*P* < 0.001). Renal biopsy reports were available for 99 CKD patients with DM (Table 4). Thirty-five (35.4%) of these patients were diagnosed with diabetic nephropathy and two (2.0%) were diagnosed with diabetic nephropathy with IgA nephropathy (IgAN); together, these patients accounted for 37.4% of all CKD patients with DM patients who underwent renal biopsies. Of the remaining CKD patients with DM who underwent biopsies, 20 (20.2%) had IgAN, 17 (17.2%) had membranous nephropathy (MN), and 9(9.1%) had mesangial proliferative glomerulonephritis. In CKD patients without DM, IgAN was the most common disease type (48.93%), followed by MN (14.36%).

**Table 4:** Pathological diagnoses of CKD patients with and without DM

Pathological diagnosis	(A) Case number (*n* = 99)	Percentage (%)	(B) Case number (*n* = 1024)	Percentage (%)
DN	35	35.4	0	0
IgAN	20	20.2	501	48.93
MN	17	17.2	147	14.36
MsPGN	9	9.1	95	9.28
DN+IgAN	2	2	0	0
Other types	16	16.1	281	27.43

*P* < 0.001. A: CKD with DM group, B:CKD without DM group, DN: diabetic nephropathy, IgAN: IgA nephropathy, MN: membranous nephropathy, MsPGN: mesangial proliferative glomerulonephritis, DN + IgAN: diabetic nephropathy with IgA nephropathy.

## DISCUSSION

DM is a leading cause of CKD, and approximately 20–30% of patients with type II DM suffer from moderate to severe impairments of renal function. An estimated 21.3% of DM patients in Chinese urban areas have CKD [5]. About 30% of American adults with DM have elevated spot urine albumin excretion readings of over 30mg/g creatinine, and 19.3% have eGFRs below 60 mL/min/1.73 m^2^ [8]. The clinicopathological characteristics of CKD patients with DM are even more complicated.

Here, we examined clinical data from the largest existing cohort of Chinese CKD patients. We found that the incidence of anemia was higher in CKD patients with DM than in patients with CKD alone. Similar results have been reported in other studies [9–11]. This higher incidence of anemia in CKD patients with DM might be associated with urinary albumin excretion, chronic inflammation, glomerular hyperfiltration, oxidative stress, use of angiotensin-converting enzyme inhibitors/angiotensin receptor blockers, loss of peritubular capillaries, up regulation of the local renin–angiotensin system, effects of autonomic neuropathy, and microvascular damage [12–16].

The incidence of hyperlipidemia was also higher in CKD patients with DM than in those without DM, which is in agreement with a previous finding that dyslipidemia was common, and a major risk factor for cardiovascular complications, in CKD patients with DM [17]. Chronic hyperglycemia in DM patients might contribute to many common endothelial injuries. In addition, reduced lipoprotein lipase activity might increase triglyceride and low-density lipoprotein cholesterol levels, and reduce high-density lipoprotein cholesterol levels, in DM patients [18]. Hypoalbuminemia was more common in CKD patients with DM than in those without DM as well, possibly due to increased urinary albumin excretion in patients with both CKD and DM that can lead to serum albumin leakage.

Both systolic blood pressure and the percentage of patients with hypertension-related complications were higher in CKD patients with DM than in those without DM, which was consistent with a study of Japanese patients [19]. Increased blood volume and vascular resistance resulting from insulin resistance in DM might contribute to the development of hypertension [20]. In patients with both DM and CKD, additional factors, such as sympathetic stimulation, renin–angiotensin–aldosterone system activation, water–sodium retention, and reductions in levels of vasoactive substances, may contribute to elevated blood pressure [21].

In this study, patients with both CKD and DM had a higher incidence of cardiovascular disease than CKD patients without DM, which is consistent with a previous study [22]. Hypertension and dyslipidemia might contribute to cardiovascular disease in CKD patients with DM. Additionally, chronic inflammation, insulin resistance, endothelial dysfunction, and atherogenic lipoprotein variation play important roles in the development of cardiovascular complications [23].

In our analysis of factors that might be associated with co-occurrence of CKD and DM, age, anemia, hypoproteinemia, hypertension, cardiovascular disease, smoking, BMI, and 24-h urinary protein level were identified as possible contributors. However, these factors cannot be confirmed as risk factors for diabetic kidney disease due to limitations of the cross-sectional design used in this study. Previous studies have suggested that age, smoking, BMI, and proteinuria are risk factors for the development of diabetic kidney disease in different patient populations [18, 24, 25].

Though anemia was more common in CKD patients with DM, fewer of these patients took anti-anemic drugs compared to CKD patients without DM. In addition, less than 50% of patients in either group were prescribed antilipemic, cardiovascular, cerebrovascular, or anti-anemic drugs. These drugs may therefore be under-prescribed in CKD patients, perhaps contributing to complications and could delaying improvements in renal function. Finally, although 87.31% and 89.97% of patients were prescribed hypotensive drugs, some patients who were not receiving these treatments might have benefited from them.

Co-occurrence of CKD and DM does not always lead to the development of diabetic nephropathy. In this study, of the 99 CKD patients with DM who had undergone a renal biopsy, only 35 (35.4%) had diabetic nephropathy; 62 (60.6%) patients had non-diabetic renal disease (NDRD) and 2(2%) had NDRD with diabetic nephropathy. In a previous study, 45.5% of biopsied type 2 DM patients were diagnosed with NDRD [26]. Another retrospective study in a southern Chinese population reported an NDRD diagnosis rate of 49% (54/110) in CKD patients with type II DM [27]. The NDRD rate in other countries ranges between 10.0 and 93.5% [27]. In the CKD patients without DM included in this study, IgAN (20.2%) was the most common pathological type in NDRD patients, followed by MN (19.2%). Similar findings were reported in another study of NDRD patients, in which the most prevalent pathologic types were IgAN (43.5%) and MN (14.5%) [28]. At Xiangya Hospital, 34% of NDRD patients exhibited IgAN; MN was the second most common type (22%) [26]. However, some studies report slightly different results. For example, MN was the most commonly-observed type (26.2%), followed closely by IgAN (24.6%), in one report [27].

In general, NDRD is relatively common in CKD patients with DM, and renal biopsies are therefore crucial for correctly diagnosing and monitoring renal diseases. However, out of the 635 Chinese CKD patients with DM included in this study, only99 (18.38%) had undergone a renal biopsy. Clinicians in China should perform renal biopsies more often to help identify etiologies of CKD more effectively, thus avoiding misdiagnosis and mistreatment. The results of this cross-sectional study should be explored further in prospective studies of CKD patient populations.

## MATERIALS AND METHODS

### Study participants

3499 pre-dialysis CKD patients between the ages of 18 and 74 were enrolled in the C-STRIDE cohort between November 2011 and April 2016. C-STRIDE is a multicenter cohort of Chinese CKD patients from 39 clinical centers located in 28 cities in 22 provinces of China. Briefly, patients within the age range with specific estimated glomerular filtration rates (eGFR) were included, regardless of etiology. Severe heart failure, systemic inflammatory illness, autoimmune disease, and some other factors served as exclusion criteria; C-STRIDE criteria have been described in detail previously [7]. C-STRIDE patients with DM identified based on the World Health Organization (1998) definition [29] were included in the CKD with DM group, while CKD patients without DM were included in the CKD without DM group.

The study was approved by the ethics committees of all 39 centers involved and was conducted in accordance with the principles contained within the Declaration of Helsinki. All participants gave written informed consent before data collection.

### Data collection

Data were collected on demographic information, clinical characteristics, laboratory examinations, complications, and concomitant medication. Renal biopsy reports were collected for enrolled patients who had undergone a renal biopsy. Demographic and clinical information and laboratory examinations included gender, age, education level, body mass index (BMI), systolic blood pressure, diastolic blood pressure, eGFR (using the Chronic Kidney Disease Epidemiology Collaboration creatinine equation), smoking and drinking history, and 24 h urine albumin.

### Variable definition

Hypertension was defined by systolic blood pressure ≥140 mmHg, diastolic blood pressure ≥90 mmHg, and/or use of antihypertensive drugs within the past 2 weeks. Hyperlipidemia was defined by total cholesterol ≥6.22 mM, triglycerides ≥2.26 mM, high-density lipoprotein <1.04 mM, and/or use of antilipemic drugs within the past 2 weeks. Anemia was defined by hemoglobin <120 g/L for males and <110 g/L for females and/or use of erythropoietin, chalybeate, or another anti-anemic treatment within the past 2 weeks. Hypoalbuminemia was defined by plasma albumin <30 g/L or vascular diseases, including cardiovascular disease, cerebrovascular disease, and peripheral angiopathies. Antihypertensive, antilipemic, anti-anemic, and vascular disease drugs were considered when collecting information on drug use. Frequency of alcohol use over the past year was scored as follows: “Occasionally” indicates use 1–3 times/month and 1–2 times/week; “Frequently” indicates 3–5 times/week, almost once/day, and more than once/day. Angiotensin-converting enzyme inhibitors, angiotensin receptor blockers, calcium antagonists, diuretic hydragogue, α-acceptor blocker, β-acceptor blocker, αβ-acceptor blocker, and centrally-acting antihypertensive treatments were considered hypotensive drugs. Statins and fibrates were considered antilipemic drugs. Ferralia, folacin, and erythropoietin were considered anti-anemic drugs. Antiplatelet and anticoagulant drugs were considered vascular drugs.

### Statistical analyses

Continuous variables are presented as means ±SD or as medians with interquartile ranges, as appropriate. *T*-tests or Mann–Whitney nonparametric tests were used for comparisons between groups. Categorical variables are presented as frequencies and percentages and were compared using chi square tests.

To mitigate the effects of missing data, we performed multiple imputation for missing data values using a fully conditional specification method in SAS. For each patient with missing data, all available data values were included as predictors in the imputation procedure for the prediction of missing data values; the original scale or categories were used before being further categorized in the regression analysis. We used a regression method to impute missing values for continuous variables that were normally distributed, a predictive mean matching method for continuous variables that were not normally distributed, and a logistic regression method for categorical variables of a binary or ordinal nature. Five imputed data sets were generated and analyzed separately, and the results obtained from the separate complete data sets were then combined.

We analyzed associations between demographic, clinical, and laboratory data from the CKD with DM group and the relevant covariates using logistic regression models. Independent variables were screened first by using variables identified in previous studies that might be closely related to CKD with DM as independent variables in the regression analysis. The independent variables were then screened based on experts’ clinical and research experience. Univariable and multivariable adjusted odds ratios (ORs) with 95% CIs are reported. The following covariates were included in the multivariable logistic regression models: age, sex, education completed, 24 h urinary protein, C-reactive protein, total protein, alkaline phosphatase, CKD stage1-4, BMI, hypoalbuminemia (yes vs. no), anemia (yes vs. no), hypertension (yes vs. no), hyperlipidemia (yes vs. no), hyperuricemia (yes vs. no), vascular disease history (yes vs. no), malignant tumor (yes vs. no), urologic surgical procedures (yes vs. no), smoking (yes vs. no), and alcohol use.

Multivariable logistic regression analysis was used to identify factors related to CKD with DM. Results from regression analysis are presented as odds ratios with 95% confidence intervals. *P-*values < 0.05 were considered statistically significant. SAS statistical software (ver. 9.4; SAS Institute, CA, USA) was used for statistical analyses.
